# Cognitive impairment in early MS: contribution of white matter lesions, deep grey matter atrophy, and cortical atrophy

**DOI:** 10.1007/s00415-020-09841-0

**Published:** 2020-04-23

**Authors:** Christina Engl, Laura Tiemann, Sophia Grahl, Matthias Bussas, Paul Schmidt, Viola Pongratz, Achim Berthele, Annkathrin Beer, Christian Gaser, Jan S. Kirschke, Claus Zimmer, Bernhard Hemmer, Mark Mühlau

**Affiliations:** 1 Department of Neurology, Klinikum rechts der Isar, School of Medicine, Technical University of Munich, Ismaninger Str. 22, 81541 Munich, Germany; 2 TUM Neuroimaging Center, Klinikum rechts der Isar, School of Medicine, Technical University of Munich, Ismaninger Str. 22, 81541 Munich, Germany; 3 Department of Psychiatry and Department of Neurology, Jena University Hospital, Jena, Germany; 4 Department of Neuroradiology, Klinikum rechts der Isar, School of Medicine, Technical University of Munich, Ismaninger Str. 22, 81541 Munich, Germany; 5 Munich Cluster for Systems Neurology (SyNergy), Feodor-Lynen-Str. 17, 81377 Munich, Germany

**Keywords:** Cognitive impairment, Multiple sclerosis, White matter lesion, Thalamus

## Abstract

**Background:**

Cognitive impairment (CI) is a frequent and debilitating symptom in MS. To better understand the neural bases of CI in MS, this magnetic resonance imaging (MRI) study aimed to identify and quantify related structural brain changes and to investigate their relation to each other.

**Methods:**

We studied 51 patients with CI and 391 patients with cognitive preservation (CP). We analyzed three-dimensional T1-weighted and FLAIR scans at 3 Tesla. We determined mean cortical thickness as well as volumes of cortical grey matter (GM), deep GM including thalamus, cerebellar cortex, white matter, corpus callosum, and white matter lesions (WML). We also analyzed GM across the whole brain by voxel-wise and surface-based techniques.

**Results:**

Mean disease duration was 5 years. Comparing MS patients with CI and CP, we found higher volumes of WML, lower volumes of deep and cortical GM structures, and lower volumes of the corpus callosum (all corrected *p* values < 0.05). Effect sizes were largest for WML and thalamic volume (standardized* ß* values 0.25 and − 0.25). By logistic regression analysis including both WML and thalamic volume, we found a significant effect only for WML volume. Inclusion of the interaction term of WML and thalamic volume increased the model fit and revealed a highly significant interaction of WML and thalamic volume. Moreover, voxel-wise and surface-based comparisons of MS patients with CI and CP showed regional atrophy of both deep and cortical GM independent of WML volume and overall disability, but effect sizes were lower.

**Conclusion:**

Although several mechanisms contribute to CI already in the early stage of MS, WML seem to be the main driver with thalamic atrophy primarily intensifying this effect.

## Introduction

Cognitive impairment (CI) is a common symptom in multiple sclerosis (MS). The neuropathological substrates of MS-related CI, and hence potential therapeutic targets, are still controversial [1]. One of the earliest magnetic resonance imaging (MRI) studies on CI in MS [2] reported a strong relationship between CI and white matter lesions (WML) compatible with the classic idea of a disconnection syndrome [3]. In later MRI studies, relations of CI to deep and cortical grey matter (GM) atrophy were reported [4–11]. When we, to the best of our knowledge, reviewed the ten structural brain MRI studies on CI in MS, with the highest numbers of CI patients included [4–13], we observed inconsistent results with regard to the contribution of WML load and GM atrophy to CI in MS. This seems well conceivable given the variety of methods used which hampers comparability. Some studies found only a contribution of WML [12, 13]. In these two studies, high-resolution structural images were not available, so that changes in GM, i.e., atrophy, may have been missed. Other studies identified GM atrophy as the main driver of CI in MS. One study found an association of deep and cortical GM atrophy through voxel-wise analysis of high-resolution T1-weighted images at 3 T [4], whilst WML volume did not differ between groups. However, patients groups were heterogeneous comprising patients with relapsing–remitting (*n* = 22), secondary progressive (*n* = 29), and primary progressive (*n* = 22) MS, which may have resulted in a high variance with regard to WML volume lowering statistical power. In another two studies, primarily thalamic atrophy [5, 6] was related to CI in MS. In one of the two studies, the methodology was focused on the thalamus. In a complex statistical model, only thalamus measures explained CI; of note, raw values of normalized brain volume, deep GM, and WML volume were also significantly different between groups [5]. In the other of the two studies, healthy controls were compared to patients with PPMS which showed lower cognitive test scores. This setting required a correlation of test performance with MRI-based measures only in the patients. Again, in a statistical model including several MRI parameters (stepwise linear regression), only thalamic volume showed a significant effect, whilst raw values of other parameters differed as well [6]. Further studies reported contributions of both WML load and GM atrophy [7–11]. However, in some of these studies, groups of patients with different degrees of CI also differed in other characteristics such as disease duration and EDSS, which was not accounted for statistically, so that results do not necessarily demonstrate differential contributions to CI [7, 8]. The same applies to a study on a large group of MS patients in which 43% were classified as CI and in which voxel-wise correlations of GM with scores of different tests were performed across the whole group [10]. In a study using high-resolution MRI, CI was related to WML and a loss of deep GM and, to a small degree, lower cortical thickness [9]. Moreover in a study on 1052 patients with MS, WML volume and brain parenchymal fraction could be analyzed. A weak correlation between CI and both MRI-based parameters was found in the early stages (< 2 years) and a strong correlation in later stages (> 15 years), whilst no differentiation between GM structures was possible. Finally, atrophy of the corpus callosum has also been related to CI, although based on a lower number of patients [14–17].

In this study, we retrospectively analyzed data from a large cohort of patients with MS primarily in early stages. All included patients underwent neuropsychological testing and standardized high-resolution MRI including FLAIR and T1-weighted sequences. These data enabled us to search for structural brain changes related to CI both at the global and regional level across the whole brain. Besides identifying brain structures, we could compare the sizes of their contributions, analyze their relation to each other, and search for differential contributions.

## Materials and methods

### Data acquisition and cognition assessment

In accordance with the Declaration of Helsinki, the study was performed in the context of our prospective observational study TUM-MS, which was approved by the local ethics committee. In the context of TUM-MS, patients are followed up annually by standardized clinical and neuropsychological examinations and brain MRI. Tests include Expanded Disability Status Scale (EDSS) and cognitive screening tests, including the Multiple Sclerosis Inventory Cognition (MuSIC) [18, 19]. MuSIC consists of 5 subtests to assess the cognitive core deficits in MS: attention and memory are tested first by ‘word list A’ (immediate recall of ten spoken words performed twice), second by ‘word list B’ (immediate recall of ten spoken words performed once, now also assessing set-shifting capacity) and third by ‘word list A delayed’ (delayed recall of wordlist A performed once later during the testing); mental set-shifting and cognitive information speed processing are tested by ‘verbal fluency’ (alternating naming of terms belonging to two different categories within one minute), inhibitory control is captured by a stroop test called ‘interference’. The MuSIC score ranges from 0 to 30; a score below 20 is considered indicative of CI which has been validated in 80 controls and 158 patients with MS [18]. In our center, the treating physician assumes cognitive preservation (CP) in the absence of any indication for CI which is routinely based on his and the patient’s impression as well as on the result of the screening battery MuSIC. In case of an indication of CI, detailed neuropsychological assessment (NPA) by an experienced neuropsychologist (LT) is recommended. During NPA, the following domains are evaluated: (a) short- and long-term memory by Verbal Learning and Memory Test (VLMT), (b) memory span and working memory (verbal and visual domain) by Wechsler Memory Scale—Revised (WMS-R), (c) information processing speed by Trail Making Test A (TMT A), (d) attention by Tests of Attentional Performance (TAP, subtests Alertness and Divided Attention) and Test of Attention D2, and (e) executive functions by Trail Making Test B (TMT B) and Regensburger Test of Word Fluency (subtests category—and letter fluency). To classify patients with NPA, raw scores of neuropsychological test results are converted to percentile ranks according to the normative population at different age levels. Normative values were adopted from established neuropsychological tests. When available, normative values for German populations were used [20–24].

### Inclusion and exclusion criteria

**Fig. 1:**
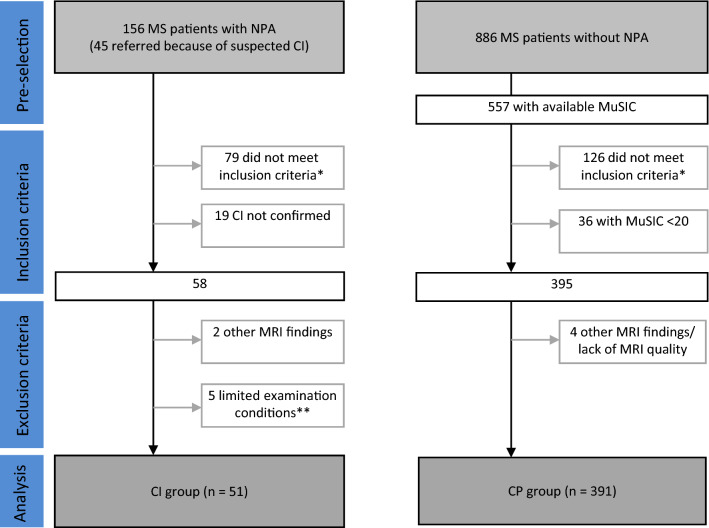
Flowchart of the selection process of patients. See text for details. (Asterisk) Inclusion criteria: brain scan with the same standardized MRI protocol, interval between the date of MRI and cognitive testing < 365 days, age between 18–69 years, disease duration < 20 years, Expanded Disability Status Score < 7.5, and exclusion of severe psychiatric comorbidity. (Double asterisk) Limited examination conditions: language barrier, 2; non-compliant, 2; aggravation, 1. *CI* cognitive impairment, *CP* cognitive preservation, *NPA* detailed neuropsychological assessment, *MuSIC* Multiple Sclerosis Inventory Cognition, *TUM-MS* prospective observational study in MS at Technical University of Munich

We studied three groups of subjects. 54 age-matched healthy controls, which had participated in other imaging studies at our institution, as well as CI and CP patients with MS. We decided to relate our measures of MS patients to those of healthy controls for reasons of quality and plausibility control. Figure 1 illustrates the selection of MS patients. In principle, we considered all patients included in TUM-MS and all patients referred to our center because of suspected CI (pre-selection). Then, we selected patients according to the inclusion criteria: availability of cognitive evaluation (either NPA or MuSIC, respectively; see below) and standardized MRI with an interval to cognitive evaluation of less than 365 days, age between 18 and 69 years, disease duration of less than 20 years, EDSS of less than 7.5, and exclusion of severe psychiatric comorbidity. For the selection of CI patients, CI had to be diagnosed by the neuropsychologist (LT) through detailed NPA. In accordance with the German guidelines on neuropsychological evaluation [25], a documented performance score of below − 1*z* in at least two domains was used as an additional inclusion criterion for quality assurance. For the selection of CP patients, we assumed CP in case the treating physician had not documented any indication of CI; further, results of the cognitive screening test MuSIC not indicating CI (i.e., values > 19) had to be available. Next, we applied the exclusion criteria, these were unusual MRI findings, violations of the MRI protocol, or limited examination conditions. From the patients with NPA, 2 were excluded due to MRI findings not typical for MS (diffuse leucencephalopathy, 1 tumefactive lesion), 5 due to limited examination conditions (2 language barriers, 2 non-compliant, 1 aggravation), and 19 as CI was not confirmed by NPA. From the CP patients, 4 were excluded due to other MRI findings (1 tumefactive lesion, 2 with artifacts, and 1 with cropped slices). Eventually, we included 51 patients with confirmed CI and 391 patients with CP before data analysis was started.

### Image acquisition and processing

All brain images were acquired on the same 3 T scanner (Achieva, Philips, The Netherlands). We used a 3D gradient-echo T1-weighted sequence (orientation, 170 contiguous sagittal 1 mm slices; field of view, 240 × 240 mm; voxel size, 1.0 × 1.0 × 1.0 mm; TR, 9 ms; TE 4 ms) and a 3D fluid-attenuated inversion recovery (FLAIR) sequence (orientation, 144 contiguous axial 1.5 mm slices; field of view, 230 × 185 mm; voxel size, 1.0 × 1.0 × 1.5 mm; TR, 10,000 ms; TE, 140 ms; TI, 2750 ms).

A detailed description of image processing was given previously [26]. In short, WML were segmented from FLAIR and T1-weighted images by the lesion growth algorithm as implemented in version 2.0.15 of the lesion segmentation tool (LST, www.statistical-modelling.de/lst.html) for SPM12 (https://www.fil.ion.ucl.ac.uk/spm). Furthermore, we applied the computational anatomy toolbox (CAT12, version 916, https://dbm.neuro.uni-jena.de/cat/) as implemented in SPM12 after lesion filling to generate normalized, modulated, and smoothed GM images (Gaussian Kernel of 8 mm) for voxel-based morphometry. For the analysis of WML location, normalized WML maps were smoothed and interpreted as WML probability maps (Gaussian kernel of 12 mm) as suggested earlier [27]. Using CAT12 segmentations and publicly available atlases as implemented in CAT12, we also determined volumes of cortical GM, thalamus, putamen, caudate nucleus, hippocampus, amygdala, nucleus accumbens, corpus callosum, global WM, and cerebellar cortex; to account for differences in head size, we extracted total intracranial volume (TIV) by a reverse brain mask method [28]. For normalization, GM volumes were not only divided by the individual TIV but also multiplied by the mean TIV (of all groups) in order not to change the order of magnitude of volumes [29]. In addition, we used CAT12 to render and analyze surface-based cortical thickness maps [30]. We recently validated this technique in MS patients [31]. For smoothing of surface images, we used a Gaussian Kernel of 8 mm. We also extracted the individual mean cortical thickness values. Finally, we studied the collinearity of extracted structural brain parameters by partial correlation analyses of all pairs of parameters. Age, sex, and EDSS were included in these partial correlation analyses.

### Group comparisons of demographic and clinical parameters as well as brain volumes

Demographic, clinical, and MRI parameters of CI and CP MS patients were compared by unpaired *t* tests (age, disease duration, EDSS, MuSIC, and brain volumes) or Chi-square test (sex). WML volume was converted to the decadic logarithm (lgWML) to approach normal distribution. For group comparisons of brain volumes, we used general linear models adjusting for age, sex, and EDSS. In these and further analyses (see below), correction for EDSS was applied as this parameter is the most established parameter of physical disability in MS; our rationale was that results surviving correction for EDSS are more likely to be related to CI, whilst results not surviving this correction may have another basis. Finally, we applied the Holm*–*Bonferroni correction to adjust for multiple testing [32]. As we extracted 12 brain volumes, uncorrected *p* values were multiplied by this number.

### Voxel-wise and surface-based analyses

To investigate whole-brain GM without any a priori hypothesis on certain brain regions, we performed voxel-wise and surface-based group comparisons by SPM12/CAT12. Age, sex, and TIV were included in these analyses. To identify regional brain changes independent from overall disability as estimated by EDSS and independent from other structural brain changes, we repeated analyses with inclusion of one additional variable. This resulted in six further analyses with the additional variable of EDSS (voxel-wise and surface-based), lgWML (voxel-wise and surface-based) volume; cerebral cortex volume (voxel-wise) and thalamic volume (surface-based). To correct for multiple comparisons across the brain, we applied threshold-free cluster enhancement (TFCE) (https://dbm.neuro.uni-jena.de/tfce/) with a corrected *p* value of < 0.05 [33]. Furthermore, we analyzed WML probability maps in a voxel-wise manner [34] to identify WML locations specifically associated with CI.

### Determinants of cognitive impairment in multiple sclerosis

Volumes showing the strongest association with CI (after correction for multiple statistical comparisons and age, sex, and EDSS) were subjected to binary logistic regression with the response variable of cognitive impairment. In such analyses, effect sizes are typically given by the exponentiation of coefficients Exp(B) corresponding to the odds ratio per unit. To allow for an intuitive interpretation of effect sizes, we *z-scaled* explanatory variables (volumes) in a way that CI had higher *z* values than CP patients. We analyzed the main effects without and with interactions.

### Software

Apart from voxel-wise and surface-based analyses, all statistical analyses were performed using version 26 of IBM SPSS; *p* values < 0.05 were considered statistically significant.

## Results

### Group comparisons of demographic and clinical parameters and brain volumes

**Fig. 2:**
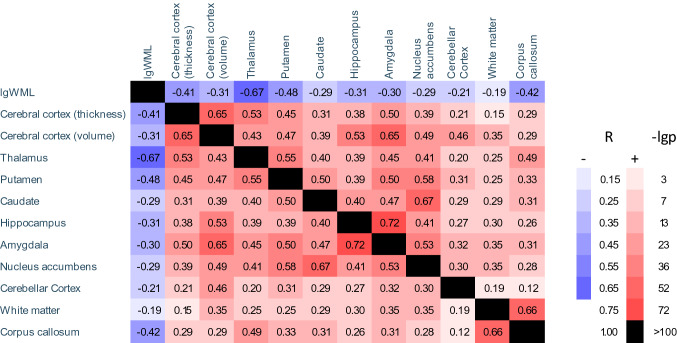
Matrix of partial correlations of brain volumes. *R* values of partial correlations of brain volumes are displayed. Control variables were age, sex, and EDSS. Significance is color-coded according to the bars on the right. *EDSS* Expanded Disability Status Scale, *lgWML* decadic logarithm of white matter lesion volume, *R* Pearson correlation coefficient, *-lgp* negative decadic logarithm of *p* value

Our healthy control group was highly comparable to our patient groups with regard to age and sex (*n* = 54, age 39 ± 5.3 years, 39 females; all *p* values of group comparisons with CP and CI patients, > 0.1). As expected, almost all brain volumes were significantly lower in both patient groups than in the control group. Therefore, we only report the results of the comparisons between patient groups with MS (i.e., CP vs. CI). CI and CP patients did not differ significantly in age, sex and disease duration (Table 1). The latter (CI 5.4 ± 5.6 years) reflected an early disease stage. Compared to CP patients, CI patients showed higher disability as measured by EDSS (3.0 ± 1.7 vs. 1.6 ± 1.1, *p* < 0.001). As expected, performance in the cognitive screening test MuSIC was much lower in the 28 CI patients in which this test battery was available (20.4 ± 5.5 vs. CP MS patients: 26.7 ± 2.9, *p* < 0.001). CI patients showed higher WML volume (lgWML 1.14 ± 0.68 vs. 0.50 ± 0.57; corresponding to WML 32.1 ± 34.8 vs. 5.6 ± 9.1 ml) and lower GM volumes (all *p* values < 0.05 after correction for multiple testing, i.e., for the 12 volumetric parameters). Of note, effect sizes were largest for lgWML and thalamic volume (standardized *ß* value 0.25 and − 0.25). Less pronounced, we also observed highly significant atrophy of the corpus callosum. Within the patients, all pairs of structural brain parameters were significantly correlated (Fig. 2). Among the parameters with the strongest association to CI, thalamic volume and WML showed the strongest correlation (*r* = − 0.67, *p* < 0.001).

**Table 1:** Demographic, clinical, and MRI parameters of cognitively preserved and cognitively impaired patients with multiple sclerosis

	CP (*n* = 391)	CI (*n* = 51, lest indicated otherwise)	Comparisons between CP and CI	
			*p* value	Standardized *beta*
Age (years)	39 ± 9.8	38 ± 9.2	0.43	0.04
Sex (male/female)	164/227	24/27	0.49	− 0.03
Disease duration (years)	4.1 ± 3.9	5.4 ± 5.6	0.11	0.10
EDSS	1.6 ± 1.1	3.0 ± 1.7	2.3E-14	**0.35**
MuSIC	26.7 ± 2.9	20.4 ± 5.5 (*n* = 28)	2.9E-22	− 0.45
TIV (ml)	1509 ± 154	1515 ± 154	0.82	0.01

Set of covariates			Age, sex, EDSS	
lgWML WML (ml) (range; median)	0.50 ± 0.57 5.6 ± 9.1 (0.02–62.3; 3.4)	1.14 ± 0.68 32.1 ± 34.8 (0.23–141; 19.5)	1.5E-7	**0.25**
Mean cortical thickness (mm)	2.494 ± 0.10	2.421 ± 0.13	5.6E-5	− 0.18

Set of covariates			Age, sex, TIV, EDSS	
Cerebral cortex (ml)	579 ± 39.1	555 ± 51.6	2.2E−5	− 0.12
Thalamus (ml)	8.52 ± 1.50	6.82 ± 2.36	1.7E−7	− **0.25**
Putamen (ml)	8.76 ± 1.00	7.98 ± 1.24	1.3E−5	− 0.20
Caudate (ml)	6.81 ± 0.92	6.22 ± 1.17	0.0004	− 0.17
Hippocampus (ml)	6.52 ± 0.57	6.22 ± 0.66	0.001	− 0.16
Amygdala (ml)	3.11 ± 0.28	2.96 ± 0.34	0.004	− 0.14
Nucleus accumbens (ml)	0.81 ± 0.09	0.77 ± 0.10	0.004	− 0.14
Cerebellar cortex (ml)	91.4 ± 8.02	86.5 ± 8.94	0.002	− 0.14
WM (ml)	515 ± 29.1	499 ± 31.1	0.001	− 0.17
Corpus callosum (ml)	18.4 ± 2.17	16.4 ± 3.24	2.6E−5	− 0.21

The two columns on the right indicate differences between cognitively impaired and cognitively preserved patients. Demographic, clinical, and basic MRI parameters were compared by unpaired t tests or Chi-square test (sex). Brain volumes (scaled by TIV) were compared by general linear models after inclusion of the covariates (control variables) of age, sex, and EDSS. Highest absolute standardized *ß* values are marked in bold. Values are given in mean ± standard deviation, lest indicated otherwise

*CI* cognitively impaired, *CP* cognitively preserved, *EDSS* Expanded Disability Status Scale, *MuSIC* Multiple Sclerosis Inventory Cognition, *TIV* total intracranial volume, *lgWML* decadic logarithm of WML, *WML* white matter lesions

### Voxel-wise and surface-based analyses

**Fig. 3:**
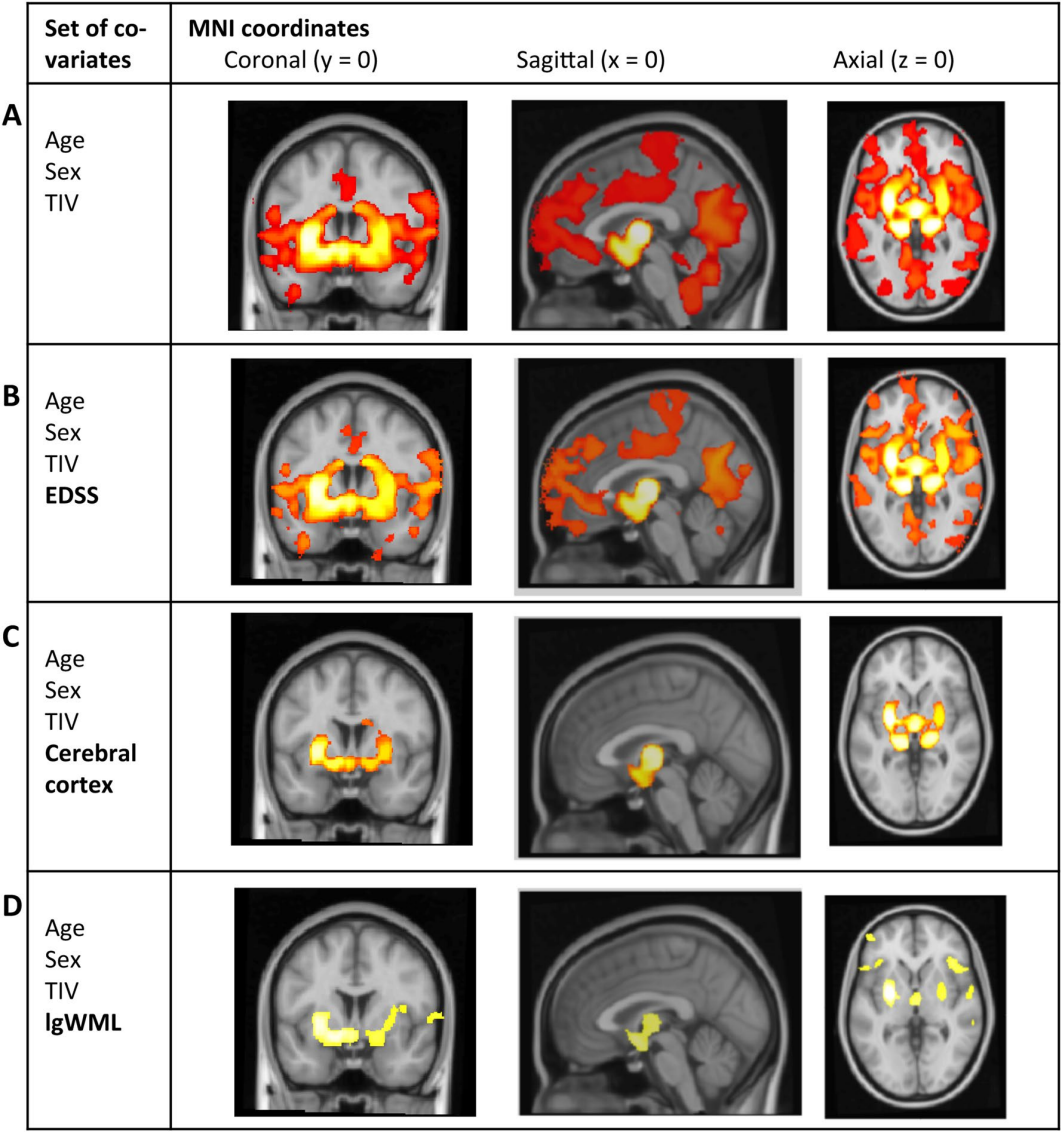
Group comparison of grey matter images. Group comparison of grey matter images between cognitively impaired and cognitively preserved patients with multiple sclerosis after adjusting for different sets of covariates. **a** Significant grey matter loss in cognitively impaired compared to cognitively preserved patients is depicted adjusting for age, sex, and TIV; **b** after additionally adjusting for disability; **c** after additionally adjusting for cerebral cortex; **d** and after additionally adjusting for white matter lesion volume. Slices are projected onto the Montreal Neurological Institute (MNI) template. Coordinates are indicated for coronal (*y*), sagittal (*x*), and axial slices (*z*). Statistical parametric maps are thresholded at *p* < 0.05 corrected (threshold-free cluster enhancement). Effects are scaled from dark red to light yellow with the latter indicating more grey matter loss. *EDSS* Expanded Disability Status Scale, *lgWML* decadic logarithm of white matter lesion volume, *TIV* total intracranial volume

**Fig. 4:**
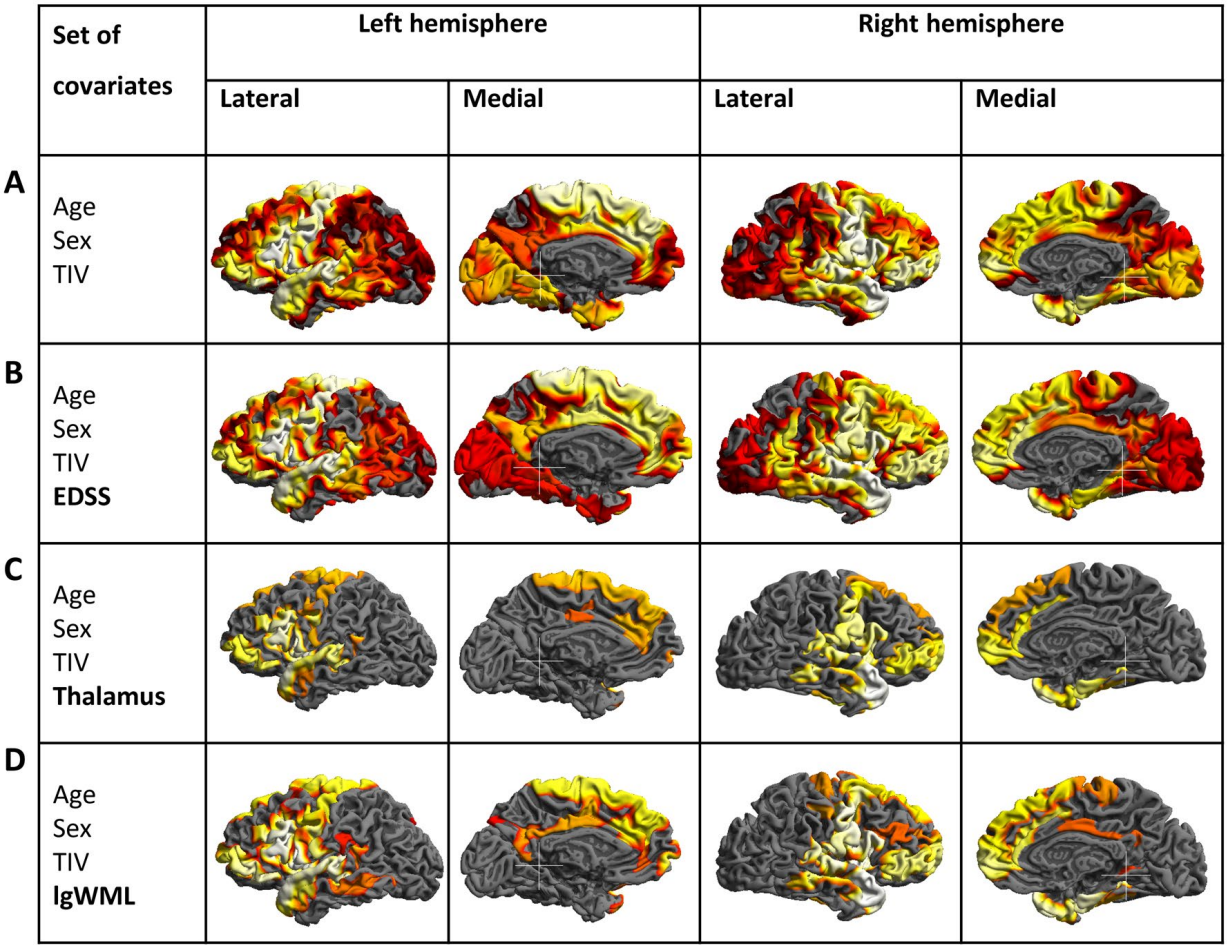
Group comparison of cortical thickness. Group comparison of cortical thickness between cognitively impaired and cognitively preserved patients with multiple sclerosis after adjusting for different covariates. **a** Significant cortical thinning in cognitively impaired compared to cognitively preserved patients with multiple sclerosis is depicted after adjusting for age, sex, TIV; **b** after additionally adjusting for disability; **c** after additionally adjusting for thalamic volume; **d** and after additionally adjusting for white matter lesion volume. The left and right hemispheres are shown separately. Increasing significance is color-coded from dark red to light yellow, the latter indicating more cortical thinning. The statistical threshold was set to *p* < 0.05 corrected (threshold-free cluster enhancement). *EDSS* Expanded Disability Status Scale, *lgWML* decadic logarithm of white matter lesion volume, *TIV* total intracranial volume

Voxel-wise analyses of GM yielded a wide-spread GM decrease in CI compared to CP most pronounced in deep GM. Adjusting for EDSS weakened but did not fundamentally change the result (Fig. 3a, b). Regional analyses of cortical thickness revealed wide-spread cortical thinning in the CI group which was only slightly weakened after adjusting for EDSS (Fig. 4a, b). Aiming at parameters exerting an independent effect on CI, we repeated the voxel-wise and surface-based analyses after inclusion of image-based covariates. In the voxel-wise analyses, thalamic atrophy remained significant after inclusion of cortical atrophy (Fig. 3c) but almost disappeared after inclusion of WML volume (Fig. 3d). Reduced cortical thickness lost significance primarily in the occipital and parietal cortex after inclusion of either thalamic volume or WML, whilst large areas of reduced cortical thickness in frontal and anterior temporal areas remained significant (Fig. 4c, d). To better visualize areas with most pronounced cortical thinning, we lowered the statistical threshold to a *p* value of < 0.05 family-wise error corrected at the voxel level revealing eight areas: bilateral parahippocampal gyrus (1 and 2), left superior frontal gyrus (3), rostral middle frontal gyrus (4), left middle superior temporal gyrus including transverse temporal gyrus (5), right precentral gyrus (6), right middle temporal gyrus (7), and right supramarginal gyrus (8).

Voxel-wise analyses of WML probability maps did not yield meaningful results. The higher WML volume of CI patients was evenly and symmetrically distributed around the ventricles reflecting the spatial WML pattern typical for MS (not shown).

### Determinants of cognitive impairment in multiple sclerosis

Here, we focused on the volumes showing the largest effects of CI, namely WML and thalamic volume. Both volumes were strongly correlated in both CP and CI patients (*r* = − 0.560 and *r* = − 0.808, respectively; both *p* < 0.001). Including the main effects of thalamic volume and WML volume in one binary logistic regression model with the response variable CI/CP (reference variable CI), only WML showed a significant contribution to CI. Including both main effects and their interaction in another model resulted in a higher pseudo-R2 value (suggesting a better model fit) and significant effects of WML volume and its interaction with thalamic volume (Table 2, upper part). Both models remained significant as described after the inclusion of overall disability estimated by EDSS (Table 2, lower part).

**Table 2:** Logistic regression on cognitive impairment

Explanatory variables: Thalamic volume, white matter lesion volume	Model with main effects only		Model with interactions and main effects	
	Pseudo R2 Nagelkerke	*p* value	Pseudo R2 Nagelkerke	*p* value
Model fit	0.23	7.1E−13	0.30	9.9E−16

	Units for *z* scaling: standard deviation	OR per SD (95% CI)	*p* value	OR per SD (95% CI)	*p* value
Thalamic volume	− 152 µl	1.30 (0.85–1.98)	0.23	0.71 (0.41–1.23)	0.22
lgWML	0.62 (≙ 4.2 ml)	2.77 (1.62–4.74)	2E−4	2.54 (1.47–4.40)	0.001
Thalamic volume * lgWML (interaction, *z* scores)	1.21	–	–	**1.92 (1.44–2.70)**	**2.1E−5**

Explanatory variables: Thalamic volume, White matter lesion volume, EDSS	Model with main effects only		Model with interactions and main effects	
	Pseudo R2 Nagelkerke	*p* value	Pseudo R2 Nagelkerke	*p* value
Model fit	0.31	1.4E−16	0.37	2.5E−19

	Units for *z* scaling: standard deviation	OR per SD (95% CI)	*p* value	OR per SD (95% CI)	*p* value
Thalamic volume	− 152 µl	1.48 (0.94–2.33)	0.09	0.78 (0.44–1.38)	0.39
lgWML	0.62 (≙ 4.2 ml)	1.91 (1.07–3.41)	0.03	1.86 (1.04–3.34)	0.04
Thalamic volume * lgWML (interaction, z scores)	1.21	–	–	**2.00 (1.44–2.79)**	**3.5E−5**
EDSS	1.3	1.98 (1.47–2.67)	6E−8	2.07 (1.50–2.85)	9.0E−6

Cognitive impairment (CI, 0; CP, 1) was used as dependent variable (reference variable CI). Thalamic and white matter lesion volumes, as well as EDSS, were used as explanatory variables. Beforehand, data were z-scaled in a way that the values of the CI group were larger than those of the CP group. We analyzed four models: (1) with inclusion of only main effects (middle columns), (2) with inclusion of both main effects and their interaction (right columns), (3–4) repetition of (1) and (2) after inclusion of EDSS

Significant interactions are given in bold

*EDSS* Expanded Disability Status Scale, *lgWML* decadic logarithm of white matter lesion volume, *OR per SD* odds ratio per unit, i.e., standard deviation, corresponding to exponentiation of coefficients, Exp(B); *SD* standard deviation

## Discussion

We aimed to study brain structures critically involved in MS-related CI by investigating a large cohort of MS patients with available cognitive testing, including confirmation of CI in 51 patients by detailed NPA, and high-resolution MRI. This enabled us not only to identify related brain structures but also to estimate their effect sizes and to analyze their differential contributions as well as their relation to each other. We will consider our study design and cohort, then discuss the results focusing on WML and thalamic atrophy, and finally, acknowledge limitations.

This study was part of our TUM-MS in-house observational study on MS planned in 2007. Data collection was performed according to a standardized protocol for regular outpatient visits. CP was assumed in patients without any indication of CI according to the treating physician who routinely considers his and the patient’s impression as well as the result of the screening battery MuSIC. For quality assurance, we further applied the commonly accepted cut-off score of the MuSIC test battery for CP [18]. Although analyzed and not confirmed in the original publication [18], this score may still be influenced by the other factors apart from CI in MS, such as educational level. Therefore, this threshold may have introduced a bias towards higher premorbid intellectual capacity in the CP group. In contrast, CI of all included patients was confirmed through detailed NPA by an experienced neuropsychologist. Although in accordance with the Germany guideline on neuropsychological evaluation [25], our criterion, again introduced in retrospect for quality assurance, of a documented performance score below − 1*z* in at least two domains is liberal compared to other studies [4–9, 11]. However, in these studies, cohorts with higher EDSS and longer disease durations were investigated (more than 10 years [4, 6–9, 11] and more than 7 years [5], respectively). Therefore, we believe that our CI patients were in the early stages of both MS and CI and that CI was milder than in patients of other studies. This may also explain why CI frequency was relatively low in our cohort compared to the frequency of 43–70% reported in the literature [35], where, again, most cohorts were in later stages of MS than our cohort. To conclude, we could analyze a large group of patients with comparatively mild but thoroughly confirmed CI, whilst we cannot fully exclude that a few patients of the even larger group of patients, classified as CP, were actually CI, which would have decreased statistical power rather than leading to false-positive results.

Comparing CI to CP patients, differences of three parameters stood out—all well in accordance with the literature: overall disability (i.e., EDSS) [12], WML volume, and thalamic atrophy [4–11]. In addition, voxel-wise analyses confirmed that thalamic atrophy is the most striking difference between CI and CP patients within brain GM, whilst none of the areas of CI-related cortical thinning showed an effect size in this order of magnitude. Although associations of both WML load and thalamic atrophy with CI in MS had been demonstrated before by further evidence [29, 36–40], we were impressed by the robustness of these effects compared to the remaining results. In addition, both parameters were strongly correlated, which had been reported before [27, 41, 42] although not consistently [43]. Against this background, we felt that further analyses focusing on these two parameters were justified. In a common model, only WML volume independently explained the occurrence of CI. Of note, the inclusion of the interaction term of WML volume and thalamic atrophy led to a better overall model fit indicating significant contributions of WML volume and its interaction with thalamic volume. In other words, thalamic atrophy seems to be less problematic than WML, whilst co-occurrence of the two is detrimental. In our opinion, the idea that thalamic atrophy is in part driven by WML through axonal transection of connecting fibers best explains our results [27, 42, 44]. Such an effect of WML on thalamic atrophy is likely to depend on overall volume, eloquence of location, and destructive power (i.e., the extent of axonal transection in a given WML volume). These three are also very likely to increase the occurrence of CI in MS. Hence, we speculate that, in our statistical model, the main effect of WML volume reflects the spatial extent of WML load, whilst the interaction of thalamic and WML volume reflects the destructiveness of WML and their eloquence of location. In contrast, we were unable to demonstrate an independent main effect of thalamic volume in our cohort of patients, who were primarily in the early stages of MS. Yet direct thalamic damage through MS-related pathology exists [45] and may become prominent in later stages.

With regard to the cerebral cortex, we observed wide-spread thinning in patients with CI compared to patients with CP. Although to a lower extent, we could relate cortical regions (primarily temporal and frontal lobe regions) more specifically to CI by correction for WML volume or overall disability (i.e., EDSS). The resulting effect was very robust but not in the order of magnitude of thalamic atrophy or WML. In MS patients in stages as early as our cohort, cortical atrophy was related to CI in only few studies [9, 46], whilst this effect seems to become more robust in later stages [4, 9, 47, 48]. Of note, a recent study demonstrated reduced cortical GM volume as the only significant MRI predictor of cognitive decline over a period of 5 years in a cohort at a later stage (compared to our cohort) with a mean symptom duration of 15 years at baseline [29]. We, therefore, speculate that the effect of cortical thinning on CI in MS is smaller in the early stages and comes more and more into play in later stages.

We also found atrophy of corpus callosum in MS patients with CI compared to CP. As volumetry was performed from normalized T1w images after filling (inpainting) of WML, this effect cannot be fully explained by volume loss through WML within the corpus callosum. Atrophy of the corpus callosum has been consistently described before but in smaller cohorts and, again, in later stages of MS [14, 15, 17]. Because of its strongest correlation with overall WM volume, we believe that the corpus callosum volume is an informative marker of WM pathology which is well conceivable given the densely packed long-range WM fibers within this WM structure. In line with this notion, MS-related changes in the tissue structure of the corpus callosum have been demonstrated by several studies using diffusion-tensor imaging techniques [16, 49–52].

We acknowledge limitations of our study. The educational level was not available in our cohort and could hence not be accounted for. The tests used for detailed NPA are not commonly used in MS, which reduces comparability of our cohort with the other cohorts. We could relate our clinical data only to measures based on the conventional structural MRI, so that we were restricted to quantification of MS pathology by volumetry of brain structures and T2-weighted hyperintense WML, which certainly does not cover the whole spectrum of MS pathology. Studies using more advanced techniques have indeed found associations with other MR-based parameters such as cortical lesions detected through double inversion recovery sequences [53, 54], or WM integrity damage detected through diffusion-tensor imaging [36, 50, 55]. Furthermore, our region-wise analyses can only detect effects spatially overlapping across subjects; it does not account for network information [56]. Hence, damage to different brain regions (across subjects but impeding the function of the same network) is not covered by our analysis.

In summary, we found a robust association of regional cortical thinning, corpus callosum atrophy, WML volume, and thalamic atrophy with CI. It seems that, in early MS, brain WML is the main driver of CI, whilst co-occurrence of thalamic atrophy intensifies the effect of WML. Yet, our data also demonstrate that, in the early stage of MS, comparatively mild CI is already associated with tissue damage in different brain compartments underlining the need to take CI as serious as physical symptoms. We believe that, for specific therapies of CI in MS, a deeper understanding is key. Studies on large cohorts, well-characterized (i.e. standardized diagnosis and quantification of CI), examined with multimodal imaging techniques, ideally longitudinally, will be necessary and probably only feasible in multicenter trials.
